# *Helicobacter pylori* antibody patterns in Germany: a cross-sectional population study

**DOI:** 10.1186/1757-4749-6-10

**Published:** 2014-04-26

**Authors:** Angelika Michel, Michael Pawlita, Heiner Boeing, Lutz Gissmann, Tim Waterboer

**Affiliations:** 1 Infections and Cancer Epidemiology (F020), Infection and Cancer Program, German Cancer Research Center (DKFZ), Im Neuenheimer Feld 280, Heidelberg 69120, Germany; 2 Department of Genome Modifications and Carcinogenesis, Infection and Cancer Program, German Cancer Research Center (DKFZ), Im Neuenheimer Feld 280, Heidelberg 69120, Germany; 3 Department of Epidemiology, German Institute of Human Nutrition (DIFE) Potsdam-Rehbrücke, Arthur Scheunert Strasse 114-116, Nuthetal 14558, Germany

**Keywords:** *Helicobacter pylori*, Immune response, Bead-based multiplex serology

## Abstract

**Background:**

*Helicobacter pylori* infection that is usually acquired in childhood and lasts for lifetime is mostly asymptomatic but associated with severe gastrointestinal disease including cancer. During chronic infection, the gastric mucosa is histologically changing. This forces *H. pylori* to permanent adaptation in its gastric habitat by expression of different proteins which might be reflected in distinctive antibody patterns.

**Methods:**

To characterize dynamics of the immune response to *H. pylori* we analysed 1797 sera of a cross-sectional study representative for the German population (age range 1–82 years) with multiplex serology, a fluorescent bead-based antibody binding assay that allows simultaneous and quantitative detection of antibodies. Fifteen recombinant, affinity-purified *H. pylori* proteins (UreA, GroEL, Catalase, NapA, CagA, CagM, Cagδ, HP0231, VacA, HpaA, Cad, HyuA, Omp, HcpC and HP0305) were used as antigens.

**Results:**

*H. pylori* seroprevalence (positivity for at least three antigens) was 48% and increased with age from 12% in children <15 years to 69% in females and 90% in males >65 years. Prevalences were highest (>83%) for Omp, VacA and GroEL. For 11 proteins, seroprevalence was higher in males than females (*P* < 0.05) from age 55 onwards. For all antigens, the median prevalence increase per age decade was stronger in males (8.4%, range 3.8-12.9%) than females (6.1%, range 3.4-10.8%). However, among seropositives the median number of antigens recognized increased from children <15 years to individuals >65 years stronger in females (*P* = 0.02). Antibody reactivities to GroEL, HyuA, CagM, Catalase, NapA and UreA also increased stronger in females (average 1.7-fold/decade, SD 0.5) than in males (1.5-fold/decade, SD 0.4).

**Conclusion:**

*H. pylori* antibody response accumulates qualitatively and quantitatively with age. This may reflect a lifelong stimulation of the immune response by chronically active infection.

## Background

More than half the world’s middle-aged population is infected with the gram-negative bacterium *Helicobacter pylori.* Overall estimate of prevalence of infection is 78% in developing and 58% in developed countries [1]. Prevalence of infection steadily declines in the industrialized Western World and emerging economies [2]. The bacterium is ingested orally and is transmitted within families mostly by the mother [3,4]. *H. pylori* infection is acquired in childhood and if untreated persists lifelong as a chronically active infection [5]. Although the majority of infections are asymptomatic, the chronic inflammatory changes of the gastric mucosa hold the risk for serious diseases of the gastrointestinal tract. Clinical manifestations begin with acute gastritis, which in a fraction of cases evolves to chronic atrophic gastritis. Gastric ulcer develops in 10% of infected individuals, and gastric adenocarcinoma in 2% and rarely mucosa-associated lymphoid tissue (MALT) lymphoma is induced [6]. It is still under debate if and when to screen and whom to treat for *H. pylori* to reach maximum benefit [7,8]. For development of disease a permanent gastric inflammatory response to infection appears to be essential [9] and inflammation is enforced by a complex interplay of bacterial virulence factors, host cofactors (such as mediators of inflammation), genetic predispositions (such as IL-1ß polymorphisms), and dietary factors [10].

The *H. pylori* genome is of high plasticity and genomic changes such as recombination, mutation and uptake even of exogenous DNA modulate the interaction with the host and adapt the bacterium to environmental changes that occur with duration of infection and stage of disease [10,11]. These interactions with the host might change the complex immune response with age and might be reflected in specific antibody patterns which have so far rarely been investigated in the context of age and gender.

We have recently developed *H. pylori* multiplex serology [12]. In contrast to conventional serological diagnosis of infection, multiplex serology simultaneously quantifies antibodies directed against arrays of protein antigens [13]. Bacterially expressed, affinity-purified glutathione-*S* transferase (GST) fusion proteins presenting conformational epitopes [14] are used as antigens. They are bound to individual sets of fluorescent polystyrene beads and antigen-loaded bead mixtures are exposed to human serum in a single reaction. For each bead set, antibodies bound to the respective antigen are quantified by streptavidin-R-phycoerythrin labelled monoclonal antibodies to human immunoglobulin. Multiplex serology allows analysis of 2000 sera per day for antibodies to up to 100 different antigens and thus provides a high-throughput platform for detection of antibody patterns in large epidemiological studies.

Using *H. pylori* multiplex serology [12], we have previously identified antibodies to HcpC and GroEL as new independent virulence factors that, in combination with the established markers anti-CagA and anti-VacA, were highly predictive of chronic atrophic gastritis risk [15]. We also found anti-CagA and anti-GroEL to be independent predictors of gastric cancer in a German case–control study [16]. Antibodies to all fifteen *H. pylori* proteins were associated with gastric cancer in a Swedish population-based cancer case–control study [17] and seropositivity to six proteins (Omp, HP305, HyuA, HpaA, CagA and VacA) may be a risk marker for distal gastric cancer in the high-incidence population of China [18].

To characterize the dynamics of the immune response as reflected in age and gender specific antibody patterns to fifteen different *H. pylori* proteins in a healthy population, we analysed 1,797 German individuals of a cross-sectional study representative for the general population covering the range from 1–82 years of age [19] with *H. pylori* multiplex serology.

## Results

### H. pylori antibody response in the German population

We analysed the antibody response to fifteen *H. pylori* proteins, i.e. UreA, GroEL, Catalase, NapA, CagA, CagM, Cagδ, HP0231, VacA, HpaA, Cad, HyuA, Omp, HcpC and HP0305 in 1,797 sera of the German population covering the range of 1 to 82 years of age (Table 1). Overall *H. pylori* seroprevalence (Hp+), defined as antibody reactivity with at least four *H. pylori* proteins [12], was 48% (Table 1).

**Table 1:** Characteristics of the study population (n = 1797)

		***n*** **(%)**
Sex	Female	1040 (57.9)
	Male	757 (42.1)
Age [years]	0-14	187 (10.4)
	15-24	235 (13.1)
	25-34	377 (21.0)
	35-44	278 (15.5)
	45-54	281 (15.6)
	55-64	265 (14.7)
	65-82	174 (9.7)
*H. pylori* seropositivity	negative (Hp-)	933 (51.9)
	positive (Hp+)	864 (48.1)

Using predefined cut-off values for classification [12], *H. pylori* protein-specific antibody prevalence in all 1,797 sera was highest for Omp (54%), GroEL (47%) and VacA (46%), lowest for Cad (15%) and distributed between 25% and 35% for the other proteins (Table 2).

**Table 2:** **Prevalence of antibodies to *H. pylori *** **proteins by HP serostatus**

			**Seroprevalence (%)**			
**Protein**	**Name**^**b**^	**Cut-off [MFI]**	**All (*n*** **= 1797)**	**Hp-**^**a**^**(*n*** **= 933)**	**Hp+**^**a**^**(*n*** **= 864)**	**P**^**c**^
HP0547	CagA	3091	33	5	63	< 0.0001
HP0010	GroEL	100	47	10	86	< 0.0001
HP1564	Omp	342	54	23	88	< 0.0001
HP0887	VacA	292	46	12	83	< 0.0001
HP0305	HP0305	100	26	2	52	< 0.0001
HP0410	HpaA	100	25	8	43	< 0.0001
HP0522	Cagδ	107	35	13	60	< 0.0001
HP0695	HyuA	274	27	5	50	< 0.0001
HP1104	Cad	100	15	5	26	< 0.0001
HP0537	CagM	178	30	7	56	< 0.0001
HP0875	Catalase	487	31	9	56	< 0.0001
HP0231	HP0231	100	28	4	53	< 0.0001
HP1098	HcpC	158	34	2	69	< 0.0001
HP0243	NapA	100	27	5	51	< 0.0001
HP0073	UreA	216	33	16	51	< 0.0001

^a^classification of *H. pylori* seropositivity as antibody reactivity with at least 4 antigens; ^b^see Materials and Methods for protein full names; ^c^Fisher’s exact test, comparison of Hp- and Hp + sera.

For each *H. pylori* protein, antibody prevalence was significantly higher in Hp+ than in Hp- sera (all *P* < 0.0001, Table 2). In Hp+ sera, antibodies to Omp (88%), GroEL (86%) and VacA (83%) were most prevalent. Antibodies to Cad (26%) were least prevalent and ranged from 43% to 69% for the other eleven *H. pylori* proteins. In Hp- sera, reactions to Omp (23%), UreA (16%), Cag (13%) and VacA (12%) were most prevalent (Table 2).

### H. pylori seroprevalence varies by age and gender

Overall *H. pylori* prevalence was similar for both genders with 48% in females and 49% in males. Hp + individuals were significantly older (median: 49 years) than Hp- individuals (median: 29 years, *P* < 0.0001). However, the *H. pylori* protein-specific seroprevalences depend strongly on age and gender.

**Figure 1:**
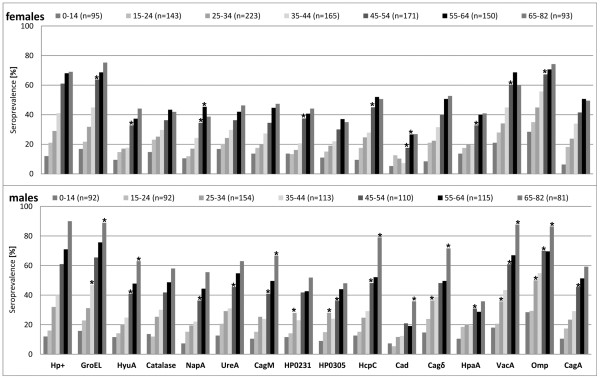
***H. pylori *seroprevalence and antigen-specific seropositivity to 15 different *H. pylori *proteins in the German population.** Seroprevalence values (%) are given stratified by age group and gender. Stars above columns indicate significant seroprevalence differences between the corresponding age group and the previous age group within gender (*P* < 0.05, Fisher’s exact test). *H. pylori* seroprevalence and all antigen-specific prevalences increased with age (*P* for trend < 0.0001, Mantel-Haenszel *χ*^2^ test) stronger in males than females.

*H. pylori* seroprevalence and *H. pylori* protein-specific prevalences were maximum in the oldest two age groups and increased significantly per age decade in males and females for all *H. pylori* proteins (all *P* for trend <0.0001, Figure 1). Overall *H. pylori* seroprevalence rose more steeply in males (13.3% per decade) than in females (10.6%) This corresponds to a mean prevalence increase per year of 1.2% for both genders. For all *H. pylori* proteins, the prevalence increase per age decade was stronger in males (median: 8.4%, range: 3.8-12.9) than in females (median: 6.1%, range: 3.4-10.8), even when the oldest age group was excluded (males median: 7.5%, range: 2.4-12.7; females median: 6.1%, range: 3.4-11.4). This effect was biggest for GroEL (12.9% in males) and lowest for Cad (3.4% in females) (Figure 1).

In the oldest age group, *H. pylori* seroprevalence and prevalences for eleven *H. pylori* proteins (CagA, GroEL, Omp, VacA, Cagδ, HyuA, CagM, Catalase, HcpC, NapA and UreA) were significantly higher in males than in females. This was also observed in the age group 35-44 years for HP0231, HP0305 and Cagδ as well as for UreA (55-64 years). As the only exception significantly higher seroprevalence in females was seen for Catalase in the age group 15-24 years (Figure 1).

### Antibody reactivities increase with age among H. pylori seropositives

**Figure 2:**
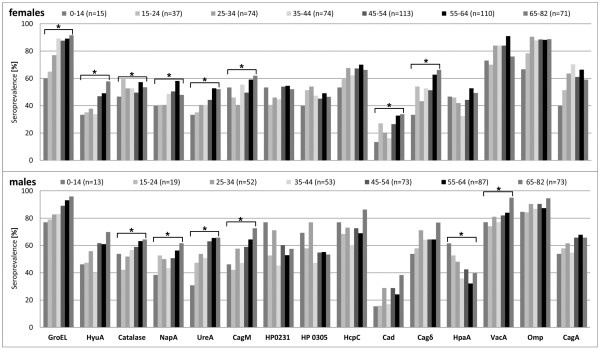
**Qualitative increase of *H. pylori *antibody responses with age in *H. pylori *seropositives (Hp+).** Seroprevalence values in Hp+ sera are stratified by age group and gender. Stars above brackets encompassing seroprevalences to a certain *H. pylori* protein indicate significant seroprevalence increases with age within gender (all *P* for trend <0.05, Mantel-Haenszel *χ*^2^ test) or decrease in case of HpaA.

Among Hp+ individuals, the antibody responses increased qualitatively and quantitatively with age. *H. pylori* protein-specific prevalences increased significantly for GroEL, HyuA, Cad, CagM, NapA and UreA in both genders and for Cagδ in females and for VacA and Catalase in males only (Figure 2). HpaA was the only antigen showing the opposing effect, a decrease from the youngest to the age group 54-65 years but this could be observed in males only (Figure 2).

**Figure 3:**
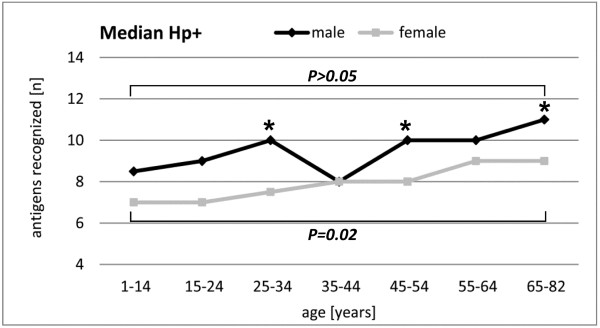
**Quantitative increase of *H. pylori *antibody responses with age in *H. pylori *seropositives (Hp+).** The median number of antigens recognized in Hp+ sera is given stratified by age group and gender. Stars indicate significant differences between genders within the same age group (*P* < 0.05, Fisher’s exact test). The difference in the median number of antigens recognized between the youngest and the oldest age group is indicated by the horizontal bracket (males: *P* > 0.05, females: *P* = 0.02; Wilcoxon two sample signed rank sum test).

Reflecting the *H. pylori* protein-specific prevalence increases, multiple seropositivity, i.e. the number of antigens recognized also increased with age for both, males and females (Figure 3). In females, the median number of antigens recognized rose from 7.0 (range 4-12) in the youngest age group to 9.0 (range 4-15) in the oldest age group (*P =* 0.02). While in males, the median started at 8.5 (range 4-13) and rose to 11 (range 5-15) in the oldest age group. The trend over time did not reach significance, but the number of *H. pylori* proteins recognized was overall higher in males than females and differed significantly between gender in the age groups 25-34, 45-54 and 65-82 years (all *P* < 0.05, Figure 3).

**Figure 4:**
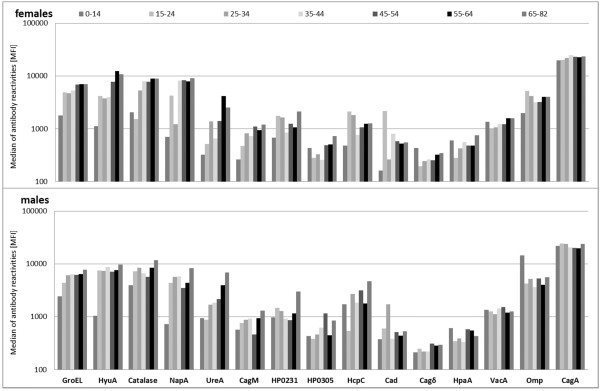
**Increase of *H. pylori *protein-specific antibody reactivities with age.** Columns show the median antibody reactivities [MFI] to 15 different *H. pylori* proteins in Hp + sera also seropositive for the respective antigens stratified by age group and gender. Antibody reactivities to GroEL, HyuA, NapA, CagM, Catalase and UreA increased from the youngest to the oldest age group with mean of 7.2-fold (SD 3.6) in females and mean 6.1-fold (SD 3.8) in males.

Antibody reactivity to several individual *H. pylori* proteins also increased with age, for both males and females. This increase was strongest among the Hp+ sera positive for GroEL, HyuA, NapA, CagM, Catalase, or UreA. The median MFI values for these six antigens increased from the youngest to the oldest age group (Figure 4) in males by mean 6.1-fold (SD 3.8) and in females by mean 7.2-fold (SD: 3.6).

## Discussion

Previous serological studies describing *H. pylori* prevalence in a general population relied on crude bacterial lysates as antigens in ELISA. In contrast, our *H. pylori* multiplex serology is based on the detection of antibodies to fifteen affinity-purified *H. pylori* proteins. Definition of *H. pylori* seropositivity is based on antibody reactivity with at least four *H. pylori* proteins. Compared to a conventional combination of screening ELISA and Western blot confirmation multiplex serology has higher specificity without loss of sensitivity [12]. A further strength of multiplex serology is its ability to allow qualitative and quantitative antibody pattern analysis [16].

The 1573 adult subjects (aged 18–82 years) analysed here are representative for the non-institutionalized adult German population [19] concerning age but not gender distribution since sera were an unbiased subset of a population-based study conducted between 1985 and 1989 with a female predominance. Children sera were collected 6 to 17 years later from hospitalised individuals without gastrointestinal disease and their ability to represent the general population is therefore limited. We cannot exclude that the longer storage times and the higher number of freeze-thaw cycles in the sera from adults might have reduced antibody reactivities, however the observed increase from children to adults in number of antigens recognized and in antibody reactivities against the individual antigens is opposite to an reducing effect of longer storage times and more freeze-thaw cycles. The increases with age among adults were observed in samples all originating from the same study with same storage and freeze-thaw cycle history.

We observed an overall *H. pylori* seroprevalence of 48%, 12% in children <15 years and 52% among adults >25 years. Several population-based studies were conducted in industrialized countries between 1982 and 1991. In 4,742 subjects from Northern Ireland (12-64 years) [20], and 2237 subjects from San Marino (23- > 70 years) [21] similar *H. pylori* prevalences of 50.5% and 51%, respectively, were reported, in contrast to lower prevalences of 35% in 3589 Danish adults (30-60 years) [22] and 38% in 273 Australian adults (20-80 years) [23].

In comparison to other studies from Germany, we observed 72% of *H. pylori* prevalence for the group 51-61 years which is higher than the 60% reported for 260 healthy adult German blood donors that were younger and might be healthier than the population analyzed here [24]. However, the 12% seroprevalence estimated here in children younger than 14 years is comparable to the 13% described for 216 children from Germany before [25].

*H. pylori* seroprevalence increased strongly with age, differing by 39% between the groups of 25-34 and 55-64 years of age. Similar age-specific prevalence estimates have been described for other developed countries [20,22,24,26] with the highest prevalence of infection in the older age groups.

The age-dependent differences in seroprevalence are either caused by a birth cohort effect [27,28] due to stronger *H. pylori* exposure in the past decades or by cumulating risk of infection with age. The prevalence of infection depends on the rate of acquisition and loss of infection. The rate of seroconversion is higher in children throughout the world [29-31] although *H. pylori* infection can be acquired at all ages [22,32]. The rate of seroconversion slows down with age in all cohorts [33,34] to small values of 0.2-1.0% per year estimated as cross sectional prevalence increase per year in adults of developed countries [30] which is slightly lower than a mean prevalence increase of 1.2% per year estimated here.

The age-dependent increases of seroprevalence were observed also for all *H. pylori* proteins reaching maximum values in males older 65 years. In this oldest age group an extraordinary steep increase in prevalence occurred for the majority of proteins in males that we did not observe in females and that lead to significant gender differences for eleven of the fifteen proteins. Previous meta-analyses also highlight a male predominance of *H. pylori* infection in 18 publications describing adult populations [35] as a global phenomenon, but not for children in ten paediatric populations [36]. In this study none of the proteins seemed to be associated with young age as a marker of childhood infection.

The conserved, surface localized lipoprotein *H. pylori* adhesion A (HpaA) does not follow the general finding of a seroprevalence increase by age in *H. pylori* seropositives. HpaA seroprevalences significantly decrease with age in males and at least do not increase in females for unknown reasons. We performed analyses stratified by HpaA status (data not shown), and in overall *H. pylori* seropositives. Antibodies to HpaA seem to be a marker for increased immune response, i.e. they are associated with higher seroprevalence to other *H. pylori* proteins and recognition of higher numbers of antigens for both genders. In contrast, HpaA serostatus does not impact seroprevalence for other *H. pylori* proteins in *H. pylori* overall seronegatives.

The major finding of our study is the observation of a qualitative increase in the immune response to *H. pylori* with age. The degree of multiple seropositivity and strength of antibody reactivity increased with age among seropositives for GroEL, HyuA**,** CagM, Catalase, NapA and UreA. These proteins are necessary for bacterial colonization and initiation of the host immune response. GroEL belongs to the chaperone family and promotes refolding of misfolded proteins under stress conditions. It seems to be associated with the adhesion of *H. pylori* to human gastric epithelial cells [37] and the induction of inflammatory responses [38]. NapA also mediates the binding of *H. pylori* to the host cell [39], activates neutrophils and monocytes and antagonizes oxidative stress [40]. Catalase is necessary for long term colonization as part of the antioxidant defence mechanisms of *H. pylori*[41]. Survival of *H. pylori* in the acidic gastric habitat is secured by its enzyme urease, composed of the two heterogeneous subunits UreA and UreB, which metabolizes urea to carbon dioxide and ammonia [42]. CagM is necessary for the translocation of the CagA protein into the host cell via the type IV secretion system [43] and HyuA belongs to the oxoprolinase family and is important for aminoacid biosynthesis [44].

This cross-sectional study does not allow distinguishing whether the increase of antibody reactivities is related to age or birth cohort effect. However our observation that this phenomenon also occurs in seropositives provides evidence that it is related to age, and immunosuppression or lower load of *H. pylori* infection in younger people as examples of birth cohort related effects are unlikely. The increase of antibody reactivities with age is opposed to the need of *H. pylori* to escape from the permanent defence of the innate and adaptive immune response. The niche of the gastric mucous layer protects *H. pylori* and makes it inaccessible to specific antibodies and thereby leaves the humoral immune response ineffective [9]. The qualitative increase of antibody reactivities with age might also reflect higher immunogenicity of bacterial antigens in individuals infected with multiple *H. pylori* strains which might be more prevalent in older age groups of the German population. Colonization with multiple *H. pylori* strains is possible [45] and change of strains may occur during chronic infection [46]. *H. pylori* adapts its genome to its host continuously by point mutation, and intragenomic and intergenomic recombination [8,11]. These mechanisms are discussed to be responsible for lifelong bacterial immune evasion and development of one’s “individual strain”. So far, *H. pylori* multiplex serology is based on antigens conserved among strains and the analysis of strain multiplicity needs to be addressed in a more comprehensive evaluation including antigens that allow for detection of strain specific antibodies.

## Conclusion

The ability of *H. pylori* multiplex serology to simultaneously and quantitatively assess antibody responses to many individual proteins has demonstrated antibody dynamics accumulating with age. This most likely reflects persistent infection and lifelong stimulation of the immune response to *H. pylori.* These findings might help to understand the balance between pathogen and host in asymptomatic individuals and could strengthen future seroepidemiological case–control studies that may allow the identification of disease-associated antibody patterns.

## Materials and methods

### *Helicobacter pylori* multiplex serology

Detection of antibodies to fifteen *H. pylori* proteins, Cad (cinnamyl-alcohol-dehydrogenase ELI3-2), Cagδ (cag pathogenicity island protein δ), CagM (cag pathogenicity island protein M), CagA (cytotoxin-associated antigen A), Catalase, HcpC (conserved hypothetical secreted protein - paralogue HcpA induces IFNγ), HP0231 (hypothetical protein HP0231), HP0305 (hypothetical protein HP0305), HpaA (neuraminyllactose-binding hemagglutinin homolog), HyuA (hydantoin utilization protein A), GroEL (chaperonin GroEL), NapA (neutrophil activating protein (bacterioferritin)), Omp (outer membrane protein), VacA (vacuolating cytotoxin), UreA (urease alpha subunit) was performed by multiplex serology [12]. The method is based on a glutathione S-transferase (GST) capture immunosorbent assay [14,47] in combination with fluorescent–bead technology as described [13]. Recombinant *H. pylori* proteins from strains 26695 and G27 were expressed in *E. coli* BL21 Rosetta (Novagen-Merck, Darmstadt, Germany) as double fusion proteins containing an N-terminal GST domain and a C-terminal tag peptide derived from the large T antigen of *simian virus 40*. The expression constructs have been described in detail [12]. Recombinant GST-*H. pylori*-tag fusion proteins from cleared bacterial lysates were loaded on spectrally distinct glutathione-casein-coupled fluorescence-labelled polystyrene beads (SeroMap, Luminex, Austin, Texas) and affinity-purified in a one-step procedure. Sera were diluted 1:50 in a serum pre-incubation buffer containing 1 mg/ml casein and 2 mg/ml total lysate protein from bacteria overexpressing GST-tag without intervening *H. pylori* sequences to block binding of antibodies directed against residual bacterial proteins, GST and the tag peptide. To suppress unspecific binding of antibodies to the beads themselves, the serum pre-incubation buffer was supplemented with 0.5% w/v polyvinylalcohol, 0.8% w/v polyvinylpyrrolidone and 2.5% v/v Superchemiblock (Millipore, Billerica, MA, USA) [48]. A monoclonal antibody directed against the C-terminal tag peptide verified the binding of the GST-*H. pylori*-tag fusion proteins to the various beads sets [14]. The differently labelled beads sets loaded with different antigens were mixed and incubated in 96 well plates with an equal volume of the serum dilutions. Antibodies bound to the beads via the GST-*H. pylori*-tag fusion proteins were stained with biotinylated goat anti-human IgA, IgM, IgG (Dianova, Hamburg, Germany) and the reporter conjugate R-phycoerythrin-labelled streptavidin. A Luminex 100 analyser identified the internal bead colour and thus the antigen carried by the bead. The quantity of bound antibodies was determined as the median reporter fluorescence intensity (MFI) of at least 100 beads per bead set per serum.

### Sera

The 1,797 sera used in this study have been described previously [19,49,50] and contained 1,573 sera from non-institutionalized donors 18 to 82 years of age (median: 41.0 years, 651 males) that were a subset (79%) of the VERA study (“Verbundstudie Ernährungserhebung und Risikofaktorenanalytik”, *n* = 1,988), which is a random subsample of the “Nationale Verzehrstudie” (NVS), a population-based nutrition survey (*n* = 23,209) performed in the West-German population between 1985 and 1989 funded by the Federal Ministry for Research and Technology. Further sera included were from patients of university hospitals in Homburg (Germany) in 2002 (*n* = 175; median age 9, range 2-18; 71 males) and Heidelberg (Germany) collected in 1991 and 1992 (*n* = 49; median age 5, range 1-10; 36 males) excluding patients with gastrointestinal disease. There was no statistically significant difference in the age structure of the 1,797 serum donors and the German standard population (*P* = 0.70).

For analyses of inter-day and inter-plate variation, a quality control (QC) panel of 31 previously characterized [12] sera containing 10 *H. pylori* negative sera (Hp-) and 21 *H. pylori* positive sera (Hp+) were included in the study.

### Assay design, data processing, and cut-off definition

Loading of glutathione-casein coupled bead sets with their respective antigens was performed in one batch. The loading efficiency was monitored via detection of the C-terminal tag of the respective antigens [14]. MFI values for the different antigens varied less than two-fold, indicating similar antigen density on the beads. Correct antigen loading was verified using previously generated data of the QC panel [12].

All sera were analysed within three consecutive days, and the QC panel was included each day. Inter-day variation was calculated from the three QC data sets of days 1, 2 and 3. Pearson correlation coefficients (R^2^) for the individual antigens were calculated from the raw MFI values and ranged from 0.96 to 0.99 (median: 0.98) for day 2 versus day 1 and from 0.88 to 0.99 (median: 0.98) for day 3 versus day 1. To correct for inter-day variation, the study data of day 2 and day 3 for each antigen were divided by the slopes of the regression lines of the respective QC data pairs. Background corrections have been previously described [12]. For all 15 antigens, antigen-specific cut-off values were applied that were previously determined in a validation study [12] from the MFI values of 20 additional sera negative in Helicobacter-R-Biopharm ELISA as mean plus 3 standard deviations excluding positive outliers [51]. Study data was normalized to this previous validation study by dividing the individual antigen specific antibody reactivity by the slopes of the regression lines of the QC data pairs of day 1 and the previous validation study.

Seropositivity for a given protein was defined as antibody reactivity greater than the antigen-specific cut-off (Table 1). *H. pylori* seropositivity (Hp+) was defined as seropositivity for at least four proteins as described previously [12].

### Statistical analyses

Statistical significance of differences in continuous variables, i.e. antibody reactivities (MFI values) and multiple seropositivity (number of antigens recognized) were analysed with the Wilcoxon two sample signed rank sum test. Fisher’s Exact test was used to test for differences in dichotomous variables, i.e. *H. pylori* seropositivity and antibody seroprevalence. Trends in seropositivity with age were estimated with the Mantel-Haenszel *χ*^2^ test on one degree of freedom. Seroprevalence increases by age were calculated using linear regression. All tests were performed two-sided. Analyses were performed with SAS software, version 9.1 (SAS Institute Inc). Unadjusted seroprevalence is reported for data stratified by age. *P*-values below 0.05 were considered statistically significant.

## Abbreviations

GST: Glutathione S-transferase; Tag: Undecapeptide from C terminus of SV40 large T-antigen; MFI: Median fluorescence intensity; Hp+/-: *H pylori* seropositive/seronegative.

## Competing interest

The authors declare that they have no competing interests.

## Authors’ contributions

Conceived and designed the experiments: MP LG. Performed the experiments: AM TW. Analyzed the data: AM TW MP. Contributed reagents/materials/analysis tools: TW HB LG MP. Wrote the paper: AM, TW, MP. Drafted the article: AM TW MP. Critically revised the article: TW HB LG MP. All authors read and approved the final manuscript.
